# 4-Hydr­oxy-3-[(2*E*)-3-(3,4,5-trimethoxy­phen­yl)prop-2-eno­yl]-2*H*-chromen-2-one

**DOI:** 10.1107/S1600536809022569

**Published:** 2009-06-20

**Authors:** Lassaad Mechi, Samar Chtiba, Naceur Hamdi, Rached Ben Hassen

**Affiliations:** a Unité de Chimie des Matériaux, ISSBAT, Université de Tunis-ElManar, 9 Avenue Dr Zoheir SAFI, 1006 Tunis, Tunisia

## Abstract

A new chalcone of the coumarin, C_21_H_18_O_7_, containing an annulated α-pyrone ring, was obtained by condensation of the borate complex of ac­yl(hydr­oxy)coumarin with trimethoxy­benzaldehyde. The structure exhibits intra­molecular hydrogen bonding between the hydroxyl oxygen and the ketonic oxygen in the coumarin group. The bicyclic coumarin fragment and the benzene ring form a dihedral angle of 17.1 (4)°. The crystal packing involves dimers inter­connected by C—H⋯O hydrogen bonding.

## Related literature

For organic non-linear optical materials (NLO) of aromatic compounds with delocalized electron systems, see: Marcy *et al.* (1995 ▶); Zhengdong *et al.* (1997 ▶). For their non-linear susceptibilities, which are larger than those of inorganic optical materials, see: Chemla & Zyss (1987 ▶) and Lakshmana Perumal *et al.* (2002 ▶), and for their optical properties, see: Sarojini *et al.* (2006 ▶). For bond-length data, see: Traven *et al.*(2000 ▶). For the exclusive annulation of the α-pyrone ring, see Traven *et al.*(2007 ▶). For charge transfer from the phenyl ring to the coumarin system, see Indira *et al.* (2002 ▶); Sun & Cui (2008 ▶).
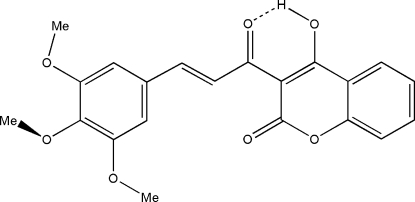


## Experimental

### 

#### Crystal data

C_21_H_18_O_7_
*M* *_r_* = 382.35
Triclinic, 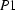

*a* = 4.1370 (2) Å
*b* = 8.1247 (2) Å
*c* = 14.4101 (2) Å
α = 74.549 (10)°
β = 85.166 (10)°
γ = 81.205 (10)°
*V* = 460.87 (4) Å^3^
*Z* = 1
Mo *K*α radiation
μ = 0.10 mm^−1^
*T* = 293 K
0.16 × 0.13 × 0.10 mm

#### Data collection

Enraf–Nonius CAD-4 diffractometer
Absorption correction: ψ scan (North *et al.*, 1968 ▶) *T*_min_ = 0.981, *T*_max_ = 0.99
3575 measured reflections
1974 independent reflections
1200 reflections with *I* > 2σ(*I*)
*R*_int_ = 0.038
2 standard reflections frequency: 120 min intensity decay: 1.1%

#### Refinement

*R*[*F* ^2^ > 2σ(*F* ^2^)] = 0.045
*wR*(*F* ^2^) = 0.136
*S* = 1.09
1974 reflections
257 parameters
3 restraints
H atoms treated by a mixture of independent and constrained refinement
Δρ_max_ = 0.12 e Å^−3^
Δρ_min_ = −0.19 e Å^−3^

### 

Data collection: *CAD-4 EXPRESS* (Duisenberg, 1992 ▶; Macíček & Yordanov, 1992 ▶); cell refinement: *CAD-4 EXPRESS*; data reduction: *XCAD4* (Harms & Wocadlo, 1995 ▶); program(s) used to solve structure: *SHELXS97* (Sheldrick, 2008 ▶); program(s) used to refine structure: *SHELXL97* (Sheldrick, 2008 ▶); molecular graphics: *PLATON* (Spek, 2009 ▶); software used to prepare material for publication: *WinGX* (Farrugia, 1999 ▶).

## Supplementary Material

Crystal structure: contains datablocks I, global. DOI: 10.1107/S1600536809022569/hg2517sup1.cif

Structure factors: contains datablocks I. DOI: 10.1107/S1600536809022569/hg2517Isup2.hkl

Additional supplementary materials:  crystallographic information; 3D view; checkCIF report

## Figures and Tables

**Table 1:** Hydrogen-bond geometry (Å, °)

*D*—H⋯*A*	*D*—H	H⋯*A*	*D*⋯*A*	*D*—H⋯*A*
O3—H4⋯O4	1.22 (6)	1.28 (6)	2.437 (5)	153 (5)
C2—H2⋯O5^i^	0.93	2.52	3.427 (6)	167
C20—H20*B*⋯O6^ii^	0.96	2.52	3.439 (8)	160
C19—H19*C*⋯O2^iii^	0.96	2.48	3.135 (7)	125
C11—H11⋯O2	0.93	2.27	2.873 (7)	122
C12—H12⋯O4	0.93	2.42	2.772 (5)	102

Symmetry codes: (i) 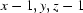; (ii) 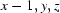; (iii) 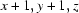.

**Fig. 1.**
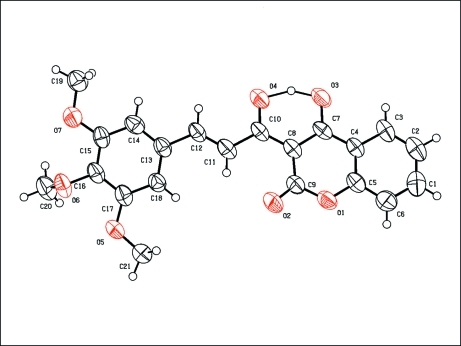
View of the title compound with atomic numbering. All atoms are shown with displacement ellipsoids drawn at the 50% probability level.

**Fig. 2.**
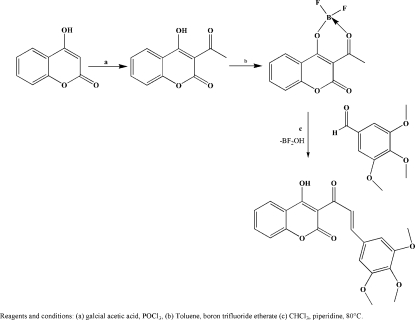
The synthesis steps of the title compund.

## supplementary crystallographic information

### Comment

The non-linear optical materials (NLO) effect in the organic molecules originates from a strong electron-donor-acceptor intermolecular interaction, delocalized π-electron system (Marcy *et al.*, 1995; Zhengdong *et al.*,1997), and also due to the ability to crystallize in non-centrosymmetric structures. Among several organic compounds reported for NLO properties, chalcone derivatives are noticeable materials for their excellent blue light transmittance and good crystallizability. They provide a necessary configuration to show NLO property with two planar rings connected through a conjugated double bond (Indira *et al.*, 2002). Substitution on either of the phenyl rings greatly influence noncentrosymmetric crystal packing. A variety of organic NLO materials, aromatic compounds with delocalized π-electron systems and a large dipole moment have been synthesized to improve the non-linear susceptibilities larger than the inorganic optical materials (Chemla *et al.*, 1987; Lakshmana *et al.*, 2002). Recently, new chalcones which can find use as promising materials in photonics industries, have been synthesized and their second-harmonic generation efficiency was studied (Sarojini *et al.*, 2006). In the present paper, we report the synthesis and the crystal structure of the trimethoxyphenyl-4-hydroxycoumarin chalcone (see Scheme). The linkage between coumarin system and phenyl ring (C13) is quite conjugated with bond lengths of C10–C11: 1.467 (6) Å, C11–C12: 1.329 (7) Å, and C12–C13: 1.471 (6) Å, suggesting that all non-hydrogen atoms between electron-donor and acceptor are highly conjugated, leading to a π-bridge for the charge transfer from phenyl ring to coumarin system (Sun *et al.*, 2008). Consequently,the C10–O4 bond (1.290 (6) Å) is elongated as compared with its mean value found in 3–Acetyl–4 hydroxycoumarin (1.253 Å) (Traven *et al.*, 2000) owing to the localization of the hydroxyl hydrogen (H4) between the ketonic oxygen O4 and the hydroxyl oxygen O3 (O3–H4: 1.22 (7) Å, O4— H4: 1.28 (7) Å) (Fig. 1). It should be noted that the C9–O2 bond length (1.210 (5) Å) is equal to its mean value 1.210 Å observed in 3–Acetyl–4 hydroxycoumarin (Traven *et al.*, 2000).

The structure study shows intramolecular and intermolecular hydrogen bonds of the type C–H···O contributing to the cohesion of the crystal.

### Experimental

It was established that the condensation of the borate complexes of acyl(hydroxy)coumarins (Traven *et al.*, 2007) with carboxylic acid anhydrides led to exclusive annulation of the α-pyrone ring. First, we prepare the borate complex of 4-hydroxycoumarin by the reaction of boron trifluoride etherate (1 g, 7.3 mmol) with the 3-acetyl-4-hydroxycoumarin (1.5 g, 7.3 mmol) in toluene (25 ml). Then the new chalcone of coumarin, containing annulated α-pyrone ring, was obtained by reaction of the borate complex of acyl(hydroxy) coumarin (1 g, 3.9 mmol) with 3, 4, 5 trimethoxyphenylaldehyde (0.78 g, 3.9 mmol) in presence of piperidine (Fig. 2). By recrystallizing the crude product in chloroform (30 ml) we tried to remove BF_2_OH from the complex and a pale yellow crystals with appropriate formula were appeared. Yield: 1.26 g (85%). mp= 466 K, IR: ν 3368 (–OH), 1716(*s*) (>C=O), 1577 (C=C), 1018(*s*) (*sym*) (C—O—C); ^1^H NMR: δ (p.p.m.): 3.74(s,3*H*,OCH_3_), 3.85(s,6*H*,OCH_3_), 7.4–8.1(m, 10H, Ar—H+ Hethyl). ^13^C NMR (ppm): 55.9(OCH_3_), 60.1(OCH_3_), 191.2(CO); 180.7 (C4); 159.5 (C2); 100.6 (C3), 126.2–136.5 (Carom); 129.6 (Cethyl1), 153.3 (Cethyl2),

### Refinement

The hydrogen atoms are fixed geometrically with the exception of the H4 where it is located from electron density difference map and is refined isotropically. In the absence of significant anomalous scattering, the absolute configuration could not be reliably determined and then the Friedel pairs were merged and any references to the Flack parameter were removed.

### Crystal data

**Table:**

C_21_H_18_O_7_	*Z* = 1
*M**_r_* = 382.35	*F*(000) = 200
Triclinic, *P*1	*D*_x_ = 1.378 Mg m^−^^3^
Hall symbol: P 1	Melting point: 466 K
*a* = 4.1370 (2) Å	Mo *K*α radiation, λ = 0.71073 Å
*b* = 8.1247 (2) Å	Cell parameters from 25 reflections
*c* = 14.4101 (2) Å	θ = 10–15°
α = 74.549 (10)°	µ = 0.10 mm^−^^1^
β = 85.166 (10)°	*T* = 293 K
γ = 81.205 (10)°	Prism, pale yellow
*V* = 460.87 (4) Å^3^	0.16 × 0.13 × 0.10 mm

### Data collection

**Table:**

Enraf–Nonius CAD-4 diffractometer	1200 reflections with *I* > 2σ(*I*)
Radiation source: fine-focus sealed tube	*R*_int_ = 0.038
graphite	θ_max_ = 27.0°, θ_min_ = 2.6°
ω/2θ scans	*h* = −5→5
Absorption correction: ψ scan (North *et al.*, 1968)	*k* = −10→10
*T*_min_ = 0.981, *T*_max_ = 0.99	*l* = −18→18
3575 measured reflections	2 standard reflections every 120 min
1974 independent reflections	intensity decay: 1.1%

### Refinement

**Table:**

Refinement on *F*^2^	Primary atom site location: structure-invariant direct methods
Least-squares matrix: full	Secondary atom site location: difference Fourier map
*R*[*F*^2^ > 2σ(*F*^2^)] = 0.045	Hydrogen site location: inferred from neighbouring sites
*wR*(*F*^2^) = 0.136	H atoms treated by a mixture of independent and constrained refinement
*S* = 1.09	*w* = 1/[σ^2^(*F*_o_^2^) + (0.0571*P*)^2^ + 0.0544*P*] where *P* = (*F*_o_^2^ + 2*F*_c_^2^)/3
1974 reflections	(Δ/σ)_max_ < 0.001
257 parameters	Δρ_max_ = 0.12 e Å^−^^3^
3 restraints	Δρ_min_ = −0.19 e Å^−^^3^

### Special details

**Table:**

Geometry. All e.s.d.'s (except the e.s.d. in the dihedral angle between two l.s. planes) are estimated using the full covariance matrix. The cell e.s.d.'s are taken into account individually in the estimation of e.s.d.'s in distances, angles and torsion angles; correlations between e.s.d.'s in cell parameters are only used when they are defined by crystal symmetry. An approximate (isotropic) treatment of cell e.s.d.'s is used for estimating e.s.d.'s involving l.s. planes.
Refinement. Refinement of *F*^2^ against ALL reflections. The weighted *R*-factor *wR* and goodness of fit *S* are based on *F*^2^, conventional *R*-factors *R* are based on *F*, with *F* set to zero for negative *F*^2^. The threshold expression of *F*^2^ > σ(*F*^2^) is used only for calculating *R*-factors(gt) *etc*. and is not relevant to the choice of reflections for refinement. *R*-factors based on *F*^2^ are statistically about twice as large as those based on *F*, and *R*- factors based on ALL data will be even larger.

### Fractional atomic coordinates and isotropic or equivalent isotropic displacement parameters (Å^2^)

**Table:**

	*x*	*y*	*z*	*U*_iso_*/*U*_eq_	
O1	0.2654 (9)	−0.1096 (4)	0.5125 (2)	0.0645 (9)	
C8	0.4138 (11)	0.1778 (6)	0.4904 (3)	0.0540 (12)	
O4	0.5686 (10)	0.4521 (5)	0.4679 (2)	0.0763 (11)	
C7	0.3170 (11)	0.2208 (6)	0.3944 (3)	0.0540 (12)	
C4	0.1785 (11)	0.0992 (6)	0.3577 (3)	0.0529 (12)	
O3	0.3412 (9)	0.3741 (5)	0.3374 (2)	0.0727 (10)	
O7	0.9754 (9)	0.6931 (4)	0.8955 (2)	0.0676 (10)	
C14	0.8343 (12)	0.5498 (6)	0.7785 (3)	0.0574 (12)	
H14	0.8904	0.6386	0.7268	0.069*	
C15	0.8677 (11)	0.5584 (6)	0.8721 (3)	0.0550 (12)	
C5	0.1553 (11)	−0.0634 (6)	0.4199 (3)	0.0563 (12)	
C11	0.5772 (13)	0.2864 (7)	0.6294 (3)	0.0620 (13)	
H11	0.5408	0.1837	0.6737	0.074*	
C18	0.6315 (12)	0.2749 (6)	0.8389 (3)	0.0550 (12)	
H18	0.5527	0.1810	0.8277	0.066*	
C17	0.6645 (11)	0.2824 (6)	0.9330 (3)	0.0574 (13)	
C10	0.5255 (12)	0.3065 (6)	0.5273 (3)	0.0557 (12)	
O6	0.8319 (9)	0.4264 (5)	1.0432 (2)	0.0717 (10)	
O2	0.5147 (10)	−0.0605 (5)	0.6284 (2)	0.0818 (12)	
O5	0.5901 (9)	0.1598 (4)	1.0150 (2)	0.0676 (10)	
C16	0.7836 (12)	0.4233 (7)	0.9499 (3)	0.0574 (12)	
C13	0.7168 (11)	0.4083 (6)	0.7613 (3)	0.0533 (12)	
C12	0.6741 (12)	0.4098 (6)	0.6607 (3)	0.0567 (12)	
H12	0.7201	0.5080	0.6139	0.068*	
C9	0.4048 (11)	0.0011 (6)	0.5495 (3)	0.0561 (12)	
C6	0.0243 (14)	−0.1878 (8)	0.3905 (4)	0.0703 (14)	
H6	0.0077	−0.2955	0.4324	0.084*	
C3	0.0705 (12)	0.1366 (7)	0.2638 (3)	0.0658 (14)	
H3	0.0857	0.2442	0.2216	0.079*	
C2	−0.0584 (14)	0.0129 (8)	0.2341 (4)	0.0771 (17)	
H2	−0.1300	0.0374	0.1718	0.093*	
C19	1.0800 (14)	0.8268 (7)	0.8172 (4)	0.0708 (14)	
H19A	1.1521	0.9128	0.8421	0.106*	
H19B	0.9008	0.8784	0.7762	0.106*	
H19C	1.2573	0.7788	0.7808	0.106*	
C21	0.4581 (15)	0.0164 (7)	1.0030 (4)	0.0723 (15)	
H21A	0.4186	−0.0602	1.0649	0.108*	
H21B	0.6104	−0.0433	0.9650	0.108*	
H21C	0.2559	0.0555	0.9708	0.108*	
C1	−0.0816 (14)	−0.1476 (9)	0.2969 (4)	0.0784 (16)	
H1	−0.1693	−0.2298	0.2762	0.094*	
C20	0.5788 (16)	0.5207 (8)	1.0864 (4)	0.0821 (16)	
H20A	0.6344	0.5155	1.1506	0.123*	
H20B	0.3794	0.4724	1.0890	0.123*	
H20C	0.5495	0.6385	1.0492	0.123*	
H4	0.478 (14)	0.441 (7)	0.387 (4)	0.086 (17)*	

### Atomic displacement parameters (Å^2^)

**Table:**

	*U*^11^	*U*^22^	*U*^33^	*U*^12^	*U*^13^	*U*^23^
O1	0.080 (2)	0.064 (2)	0.0421 (17)	−0.0060 (18)	−0.0092 (15)	−0.0010 (15)
C8	0.057 (3)	0.063 (3)	0.036 (2)	0.003 (2)	−0.0032 (19)	−0.009 (2)
O4	0.113 (3)	0.070 (2)	0.0431 (19)	−0.015 (2)	−0.0111 (19)	−0.0063 (17)
C7	0.058 (3)	0.063 (3)	0.033 (2)	0.001 (2)	0.005 (2)	−0.005 (2)
C4	0.054 (3)	0.062 (3)	0.037 (2)	0.004 (2)	−0.006 (2)	−0.010 (2)
O3	0.107 (3)	0.069 (2)	0.0371 (17)	−0.009 (2)	−0.0028 (17)	−0.0052 (16)
O7	0.081 (3)	0.067 (2)	0.055 (2)	−0.0096 (18)	−0.0085 (17)	−0.0155 (17)
C14	0.062 (3)	0.055 (3)	0.047 (2)	0.005 (2)	−0.004 (2)	−0.006 (2)
C15	0.052 (3)	0.065 (3)	0.047 (3)	0.007 (2)	−0.013 (2)	−0.020 (2)
C5	0.053 (3)	0.069 (3)	0.042 (2)	0.002 (2)	0.001 (2)	−0.013 (2)
C11	0.073 (3)	0.068 (3)	0.039 (2)	0.003 (3)	−0.006 (2)	−0.008 (2)
C18	0.066 (3)	0.051 (3)	0.044 (2)	0.001 (2)	−0.011 (2)	−0.009 (2)
C17	0.064 (3)	0.062 (3)	0.046 (2)	0.006 (2)	−0.007 (2)	−0.019 (2)
C10	0.065 (3)	0.065 (3)	0.031 (2)	0.002 (2)	−0.0040 (19)	−0.007 (2)
O6	0.084 (2)	0.086 (2)	0.0477 (18)	0.0038 (19)	−0.0186 (17)	−0.0248 (18)
O2	0.119 (3)	0.074 (2)	0.0463 (19)	−0.012 (2)	−0.031 (2)	0.0023 (16)
O5	0.093 (3)	0.069 (2)	0.0383 (16)	−0.0153 (19)	−0.0088 (16)	−0.0046 (15)
C16	0.063 (3)	0.065 (3)	0.040 (2)	0.005 (2)	−0.013 (2)	−0.012 (2)
C13	0.059 (3)	0.054 (3)	0.042 (2)	0.010 (2)	−0.009 (2)	−0.012 (2)
C12	0.069 (3)	0.062 (3)	0.035 (2)	0.000 (2)	−0.009 (2)	−0.008 (2)
C9	0.060 (3)	0.062 (3)	0.043 (3)	−0.007 (2)	−0.006 (2)	−0.008 (2)
C6	0.072 (3)	0.077 (3)	0.062 (3)	−0.014 (3)	0.008 (3)	−0.018 (3)
C3	0.066 (3)	0.090 (4)	0.040 (2)	−0.003 (3)	−0.008 (2)	−0.016 (2)
C2	0.071 (4)	0.107 (5)	0.048 (3)	0.001 (3)	−0.011 (3)	−0.016 (3)
C19	0.073 (4)	0.073 (3)	0.062 (3)	−0.006 (3)	−0.014 (3)	−0.010 (3)
C21	0.094 (4)	0.073 (3)	0.051 (3)	−0.022 (3)	0.002 (3)	−0.013 (3)
C1	0.071 (4)	0.102 (5)	0.072 (4)	−0.013 (3)	−0.006 (3)	−0.039 (3)
C20	0.097 (4)	0.095 (4)	0.054 (3)	−0.003 (3)	0.000 (3)	−0.026 (3)

### Geometric parameters (Å, °)

**Table:**

O1—C9	1.375 (6)	C17—O5	1.375 (6)
O1—C5	1.384 (5)	C17—C16	1.397 (6)
C8—C7	1.410 (6)	O6—C16	1.383 (5)
C8—C10	1.440 (7)	O6—C20	1.403 (6)
C8—C9	1.466 (6)	O2—C9	1.210 (5)
O4—C10	1.290 (6)	O5—C21	1.415 (6)
O4—H4	1.28 (7)	C13—C12	1.471 (6)
C7—O3	1.310 (5)	C12—H12	0.9300
C7—C4	1.445 (6)	C6—C1	1.392 (8)
C4—C5	1.396 (6)	C6—H6	0.9300
C4—C3	1.402 (6)	C3—C2	1.381 (8)
O3—H4	1.22 (7)	C3—H3	0.9300
O7—C15	1.372 (6)	C2—C1	1.387 (9)
O7—C19	1.431 (6)	C2—H2	0.9300
C14—C15	1.389 (6)	C19—H19A	0.9600
C14—C13	1.401 (7)	C19—H19B	0.9600
C14—H14	0.9300	C19—H19C	0.9600
C15—C16	1.406 (6)	C21—H21A	0.9600
C5—C6	1.388 (7)	C21—H21B	0.9600
C11—C12	1.329 (7)	C21—H21C	0.9600
C11—C10	1.467 (6)	C1—H1	0.9300
C11—H11	0.9300	C20—H20A	0.9600
C18—C17	1.392 (6)	C20—H20B	0.9600
C18—C13	1.398 (6)	C20—H20C	0.9600
C18—H18	0.9300		
			
C9—O1—C5	122.3 (4)	C18—C13—C14	119.8 (4)
C7—C8—C10	119.6 (4)	C18—C13—C12	122.2 (4)
C7—C8—C9	118.8 (4)	C14—C13—C12	118.0 (4)
C10—C8—C9	121.7 (4)	C11—C12—C13	127.2 (4)
C10—O4—H4	104 (3)	C11—C12—H12	116.4
O3—C7—C8	120.7 (4)	C13—C12—H12	116.4
O3—C7—C4	118.4 (4)	O2—C9—O1	115.4 (4)
C8—C7—C4	120.9 (4)	O2—C9—C8	126.4 (5)
C5—C4—C3	119.3 (4)	O1—C9—C8	118.2 (4)
C5—C4—C7	117.4 (4)	C5—C6—C1	118.5 (5)
C3—C4—C7	123.3 (4)	C5—C6—H6	120.8
C7—O3—H4	102 (3)	C1—C6—H6	120.8
C15—O7—C19	116.8 (4)	C2—C3—C4	119.7 (5)
C15—C14—C13	120.5 (4)	C2—C3—H3	120.1
C15—C14—H14	119.8	C4—C3—H3	120.1
C13—C14—H14	119.8	C3—C2—C1	120.3 (5)
O7—C15—C14	124.4 (4)	C3—C2—H2	119.9
O7—C15—C16	116.2 (4)	C1—C2—H2	119.9
C14—C15—C16	119.4 (4)	O7—C19—H19A	109.5
O1—C5—C6	116.8 (4)	O7—C19—H19B	109.5
O1—C5—C4	122.1 (4)	H19A—C19—H19B	109.5
C6—C5—C4	121.1 (4)	O7—C19—H19C	109.5
C12—C11—C10	122.3 (5)	H19A—C19—H19C	109.5
C12—C11—H11	118.9	H19B—C19—H19C	109.5
C10—C11—H11	118.9	O5—C21—H21A	109.5
C17—C18—C13	120.1 (4)	O5—C21—H21B	109.5
C17—C18—H18	120.0	H21A—C21—H21B	109.5
C13—C18—H18	120.0	O5—C21—H21C	109.5
O5—C17—C18	125.6 (4)	H21A—C21—H21C	109.5
O5—C17—C16	114.5 (4)	H21B—C21—H21C	109.5
C18—C17—C16	120.0 (4)	C2—C1—C6	121.0 (5)
O4—C10—C8	118.3 (4)	C2—C1—H1	119.5
O4—C10—C11	117.4 (5)	C6—C1—H1	119.5
C8—C10—C11	124.2 (4)	O6—C20—H20A	109.5
C16—O6—C20	115.6 (4)	O6—C20—H20B	109.5
C17—O5—C21	117.3 (4)	H20A—C20—H20B	109.5
O6—C16—C17	119.9 (4)	O6—C20—H20C	109.5
O6—C16—C15	119.9 (4)	H20A—C20—H20C	109.5
C17—C16—C15	120.2 (4)	H20B—C20—H20C	109.5

### Hydrogen-bond geometry (Å, °)

**Table:**

*D*—H···*A*	*D*—H	H···*A*	*D*···*A*	*D*—H···*A*
O3—H4···O4	1.22 (6)	1.28 (6)	2.437 (5)	153 (5)
C2—H2···O5^i^	0.93	2.52	3.427 (6)	167
C20—H20B···O6^ii^	0.96	2.52	3.439 (8)	160
C19—H19C···O2^iii^	0.96	2.48	3.135 (7)	125
C11—H11···O2	0.93	2.27	2.873 (7)	122
C12—H12···O4	0.93	2.42	2.772 (5)	102

Symmetry codes: (i) *x*−1, *y*, *z*−1; (ii) *x*−1, *y*, *z*; (iii) *x*+1, *y*+1, *z*.
